# Weight-of-evidence approach to identify regionally representative sites for air-quality monitoring network: Satellite data-based analysis

**DOI:** 10.1016/j.mex.2020.100949

**Published:** 2020-06-04

**Authors:** Nirav L Lekinwala, Ankur Bharadwaj, Ramya Sunder Raman, Mani Bhushan, Kunal Bali, Sagnik Dey

**Affiliations:** 1 Department of Chemical Engineering, IIT Bombay; 2 Interdisciplinary Programme in Climate Studies, IIT Bombay, Mumbai - 400076, Maharashtra, India; 3 Department of Earth and Environmental Sciences, IISER Bhopal; 4 Center for Research on Environment and Sustainable Technologies, IISER Bhopal, Bhopal Bypass Road, Bhauri, Bhopal – 462 066, Madhya Pradesh, INDIA; 5 Center for Atmospheric Science, IIT Delhi, Delhi - 110016, Delhi, India; 6 Centre of Excellence for Research on Clean Air (CERCA), IIT Delhi

**Keywords:** MODIS, MAIAC Algorithm, Aerosol Optical Depth, Coefficient of Divergence, Pearson Correlation Coefficient, Mutual Information, Python, CDO

## Abstract

The methodology discussed in Lekinwala et al., 2020, hereinafter referred to as the ‘parent article’, is used to setup a nation-wide network for background PM_2.5_ measurement at strategic locations, optimally placing sites to obtain maximum regionally representative PM_2.5_ concentrations with minimum number of sites. Traditionally, in-situ PM_2.5_ measurements are obtained for several potential sites and compared to identify the most regionally representative sites [4], Wongphatarakul et al., 1998) at the location. The ‘parent article’ proposes the use of satellite-derived proxy for aerosol (Aerosol Optical Depth, AOD) data in the absence of in-situ PM2.5 measurements. This article focuses on the details about satellite-data processing which forms part of the methodology discussed in the ‘parent article’. Following are some relevant aspects:

• High resolution AOD is retrieved from Moderate Resolution Imaging Spectroradiometer (MODIS) instruments aboard NASA's Aqua and Terra satellite using Multi-Angle Implementation of Atmospheric Correction (MAIAC) algorithm. The data is stored as grids of size 1200  ×  1200 and a total of seven such grids cover the Indian land mass. These grids were merged, regridded and multiplied by conversion factors from GEOS-Chem Chemical Transport Model to obtain PM_2.5_ values. Standard set of tools like CDO and NCL are used to manipulate the satellite-data (*.nc files).
• The PM_2.5_ values are subjected to various statistical analysis using metrics like coefficient of divergence (CoD), Pearson correlation coefficient (PCC) and mutual information (MI).
• Computations for CoD, MI are performed using Python codes developed in-house while a function in NumPy module of Python was used for PCC calculations.

## Graphical abstract

**Figure:**
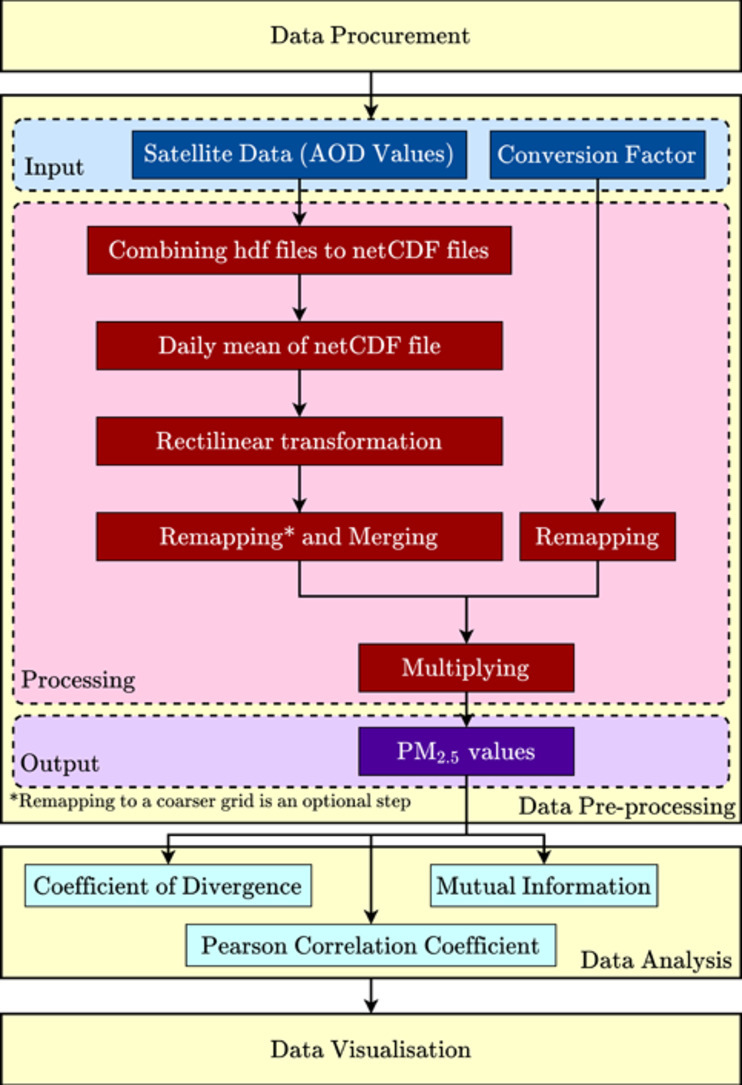

**Table:** Specifications table

Subject Area	Environmental Science

More specific subject area	Use of satellite data in Air Quality Monitoring (AQM)
Method name	Satellite-derived PM_2.5_ to establish regional representativeness using statistical metrics (CoD, PCC, MI)
Name and reference of original method	The current work focuses on implementation of application of Mutual Information [3] as a metric to capture non-linear relationship in the data. Additionally, metrics like CoD and PCC discussed in [7], is used in the study.
Resource availability	AOD data can be downloaded from https://portal.nccs.nasa.gov/datashare/maiac/DataRelease/China/ AOD to PM_2.5_ Conversion factors are not available online and were procured from Dr. Sagnik Dey and group which is based on the study [8] Other software can be downloaded from 1. Python: https://www.python.org/ 2. CDO: https://code.mpimet.mpg.de/projects/cdo 3. NCL: https://www.ncl.ucar.edu/

## Method Details

The method discussed in the article covers procurement and pre-processing of the satellite data, analysing them using metrics like Coefficient of Divergence (CoD), Pearson Correlation Coefficient (PCC) and Mutual information (MI) and visualising the spatial map of the metrics. The verification and validation of this method are presented in the parent article [5]. Further, all codes that were developed for this work are presented as supplementary material.

### Data Procurement

**Fig. 1:**
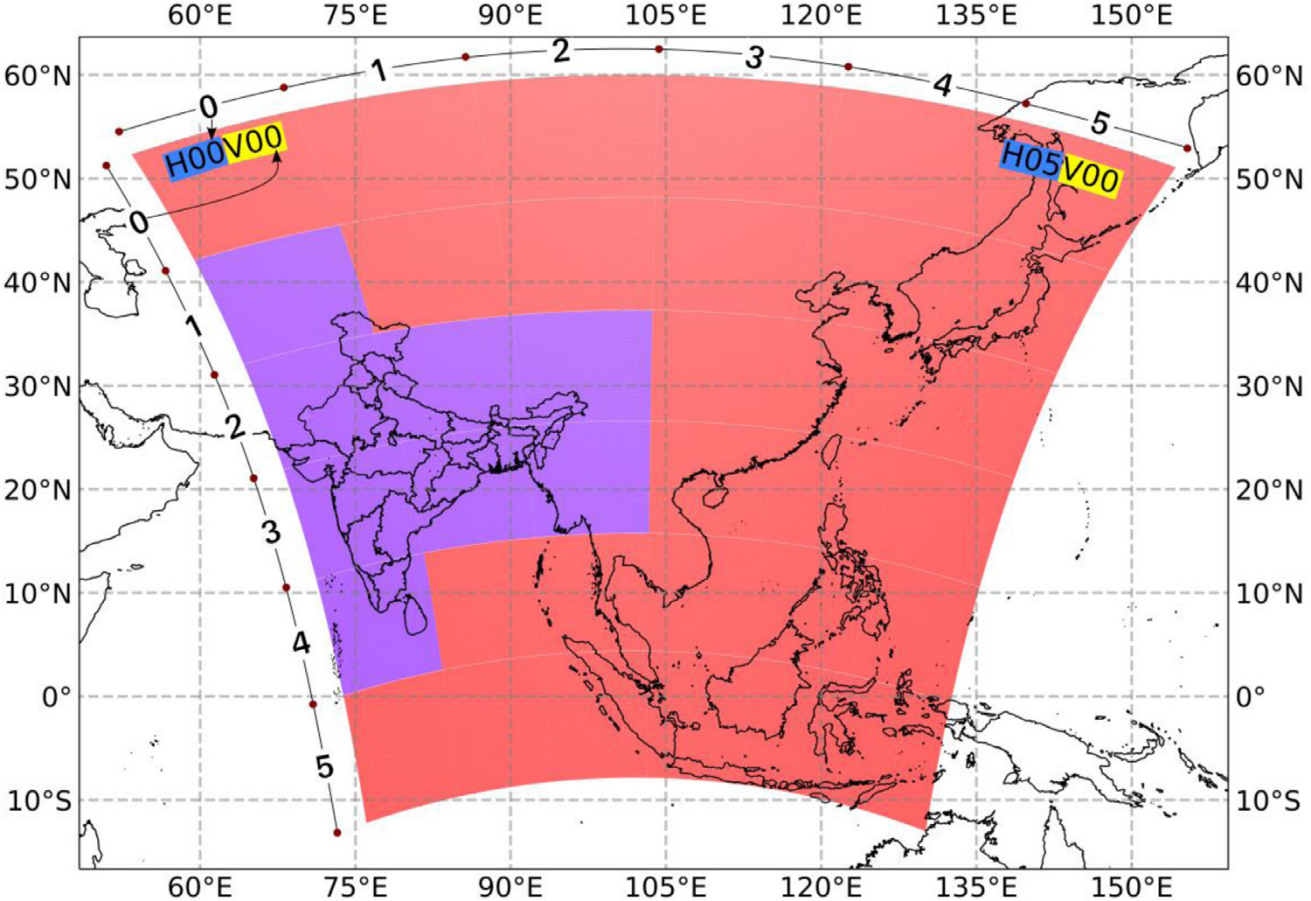
Scope of the high-resolution data available from MODIS sensor aboard NASA's Aqua and Terra satellite using MAIAC algorithm. The data in purple coloured grids are relevant for this work.

The high-resolution AOD data is freely available over the Eastern and South-Eastern Asia region and are divided into grids as shown in the Fig. 1 below. Indian landmass is covered by eight grids of size 1200km  ×  1200 km each as highlighted in Fig. 1.

The files can be downloaded from NCCS's Dataportal [1]. Grid-wise daily files of AOD values are available in the year-wise folders each containing multiple AOT files per day (multiple swaths for Aqua and Terra, max 4 files per day) with unique file names. For example, in grid h00v01 [2], the files are sorted annually; for the year 2004, files are named according to the following convention,

**Table:** 

MAIACAAOT.h00v01.20040010735.hdf	
Part in Name	Description
MAIAC	Algorithm used on the MODIS data to obtain data products [6]
A/T	NASA's Aqua/Terra satellite fitted with MODIS sensor
AOT	Aerosol optical thickness
h00v01	The grid name as shown in Figure 2
2004	Year
001	Day of the year, can range from 001 – 365/366 (depending on leap year), 001 is equivalent to January 01
0735	Time at which the satellite passed over the region
hdf	File extension

### Data pre-processing

**Fig. 2:**
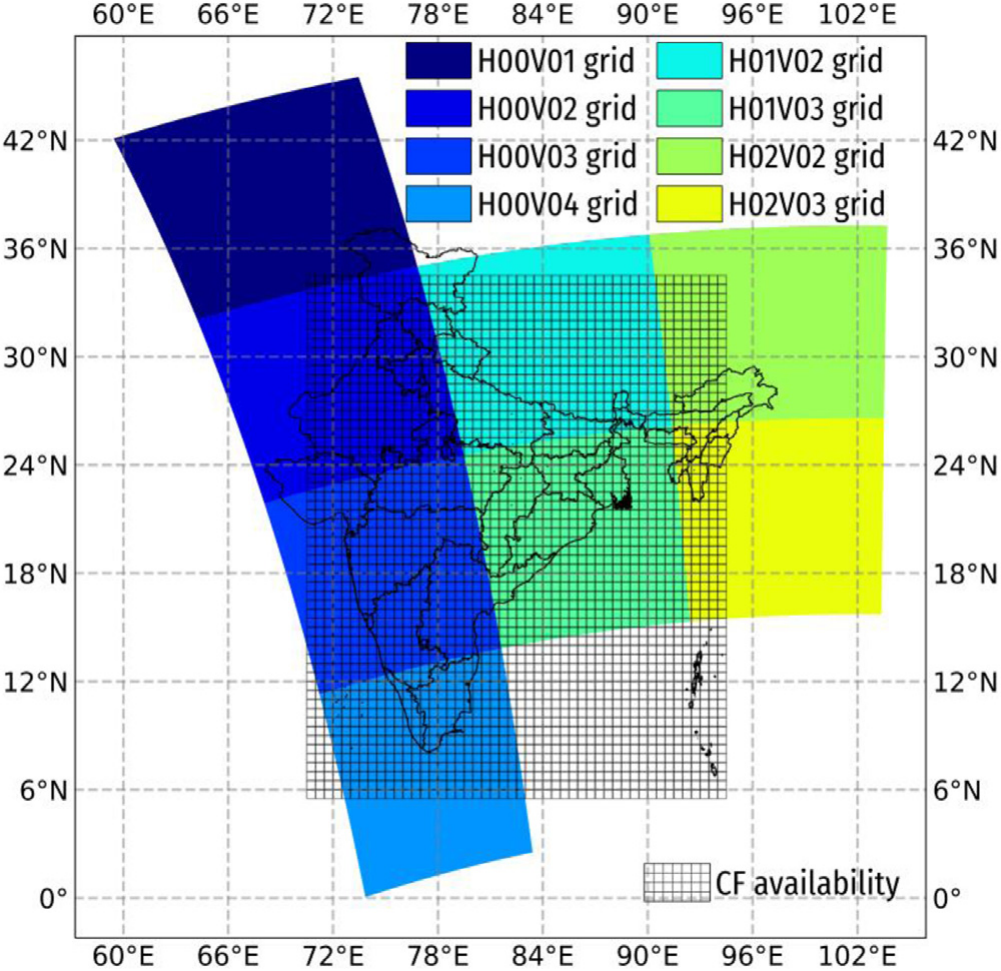
Scope of 8 grids covering India in different colors and the scope of the available conversion factor shown in mesh.

**Fig. 3:**
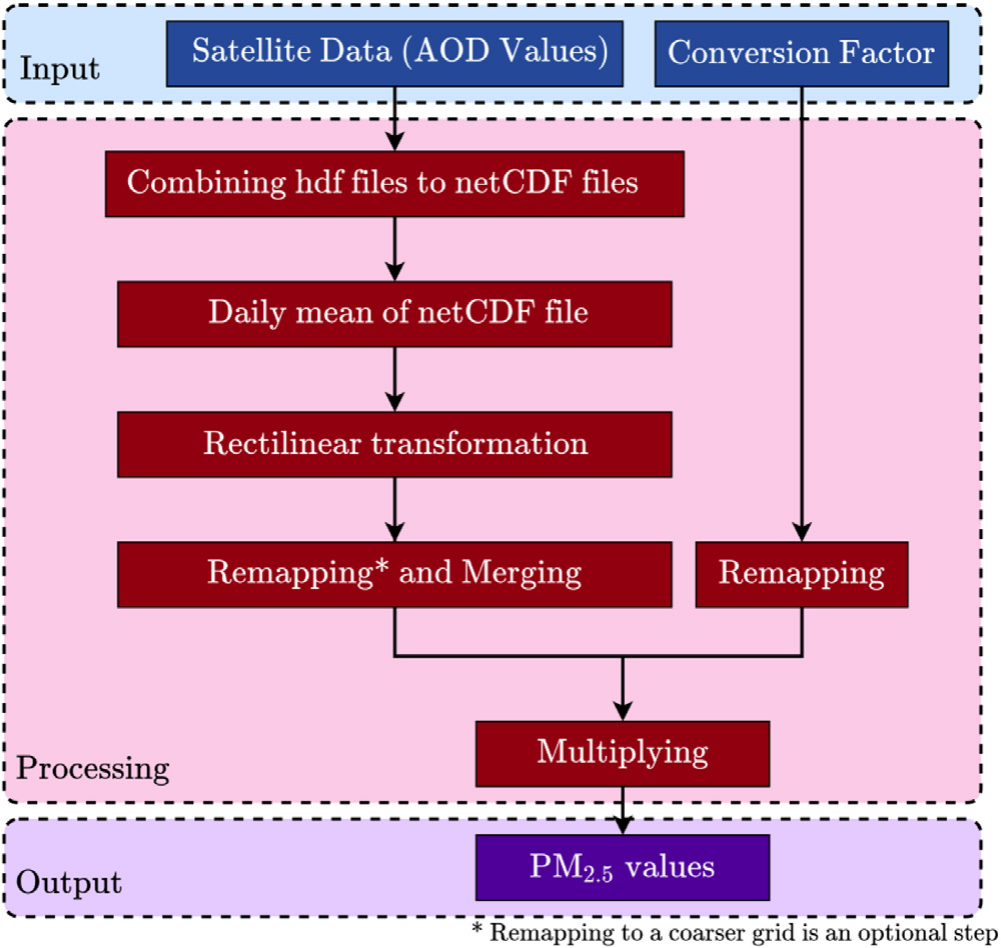
Schematic of steps involved in data pre-processing

The files for the years 2004 – 2011 were procured for the current methodology as the AOD/AOT conversion factors (CF) were available for these years. Fig. 2 shows the grids over India and the scope of availability of the CF. Fig. 3 schematically shows the data pre-processing steps involved in the methodology.

1. **Combining the hdf files to netCDF files:** An NCL code to read all the files and extract the variable Optical_Depth_055 (Aerosol optical depth at 550 nm i.e, 0.55 *μ*m) and write it to the netCDF file along with latitude, longitude and time data was developed. The code used for combining is attached with the article and is named processing_01.ncl which generates MAIACTAOT-h00v02-ANNUAL-20040010730-20113650555.nc file containing data from all hdf files.
  a. The details about the newly created netCDF file can be checked using ncdump function (as a part of netcdf-bin package on a Linux-based PC). ncdump of one file is shown below, it shows that there are 9474 time-steps available (multiple daily time instants due to multiple passes of both Aqua and Terra every day).
  b. The process of converting hdf files into netCDF files is repeated for all the grids. Bottle-neck in this process is the disk writing speed (file for each grid is about 32GB in size) which may take up to an hour per grid. For all the computations, Linux-based computer with 6 core – 12 thread i7-8700 processor, 24 GB RAM and a standard spinning hard-disk drive is used.
2. **Daily mean of netCDF file:** To make the processing easier and bring uniformity across different grids, the daily mean of the data is used, thus making total number of time instants (2922 days) same across different grids.
3. **Rectilinear transformation:** The data grids obtained from the satellite are curvilinear in nature. The daily-averaged data for different grids needs to be merged to create a consistent spatio-temporal dataset over all the time instants. In order to merge the grids, they need to be transformed from the curvilinear form to rectilinear form using code rectilinear.ncl which is later remapped using grid characteristics given in regrid.txt.
  a. The NCL code creates a blank rectilinear *.nc file.
  b. The blank rectilinear *.nc file is populated with the variables and saved separately using CDO's setgrid function.
4. **Remapping and Merging:** The merging of grid is important to ensure spatial continuity in the data and makes processing easy. Following are some points to note,
  a. To merge the different grids, a large grid covering the extent of the smaller grids needs to be created.
  b. Remapping process is computationally expensive and time consuming and thus to establish a balance between spatial resolution and computation time, for the current application, the 1 km  ×  1 km data was remapped (converted) to 1.5 km  ×  1.5 km using CDO's remapcon function.
  c. This resolution was found optimal as without losing fineness in the data, the computation time was reduced by about 2-2.5 times (about half the original time).
  d. It took about 400 hours for the Remapping to complete on a 6-core, 12-thread 4GHz Intel i7-8700 machine with 24GB of RAM.
  e. Remapping the data to the original spatial resolution is essential before merging, while Remapping it to a coarser grid is optional.
  f. During the merging process (using CDO's mergegrid function), a minimum 500 GB of storage is required for all the auxiliary files (intermediate files) which can be deleted after successful merging.
  g. In case of some issue or some error in the file, the computation can be resumed from a step before the error occurred if the auxiliary files are saved until successful completion of Remapping and merging.
5. **Remapping CF and Multiplying AOD with CF:** For the current study, the CF are obtained from the GEOS-Chem Chemical transport model which is adopted from the study of van Donkelaar et al., [8].
  a. The daily CF (2004 - 2011) are already in rectilinear form, it is easy to remap it to same spatial resolution as the AOD.
  b. The daily CF value is multiplied with the daily AOD values to obtain daily PM_2.5_ values.
  c. The multiplication is computationally expensive and is also bottlenecked by the storage speed, it may take about 4-6 hours to process about 15.4  ×  10^12^ floating point operations.

## Data Analysis

**Fig. 4:**
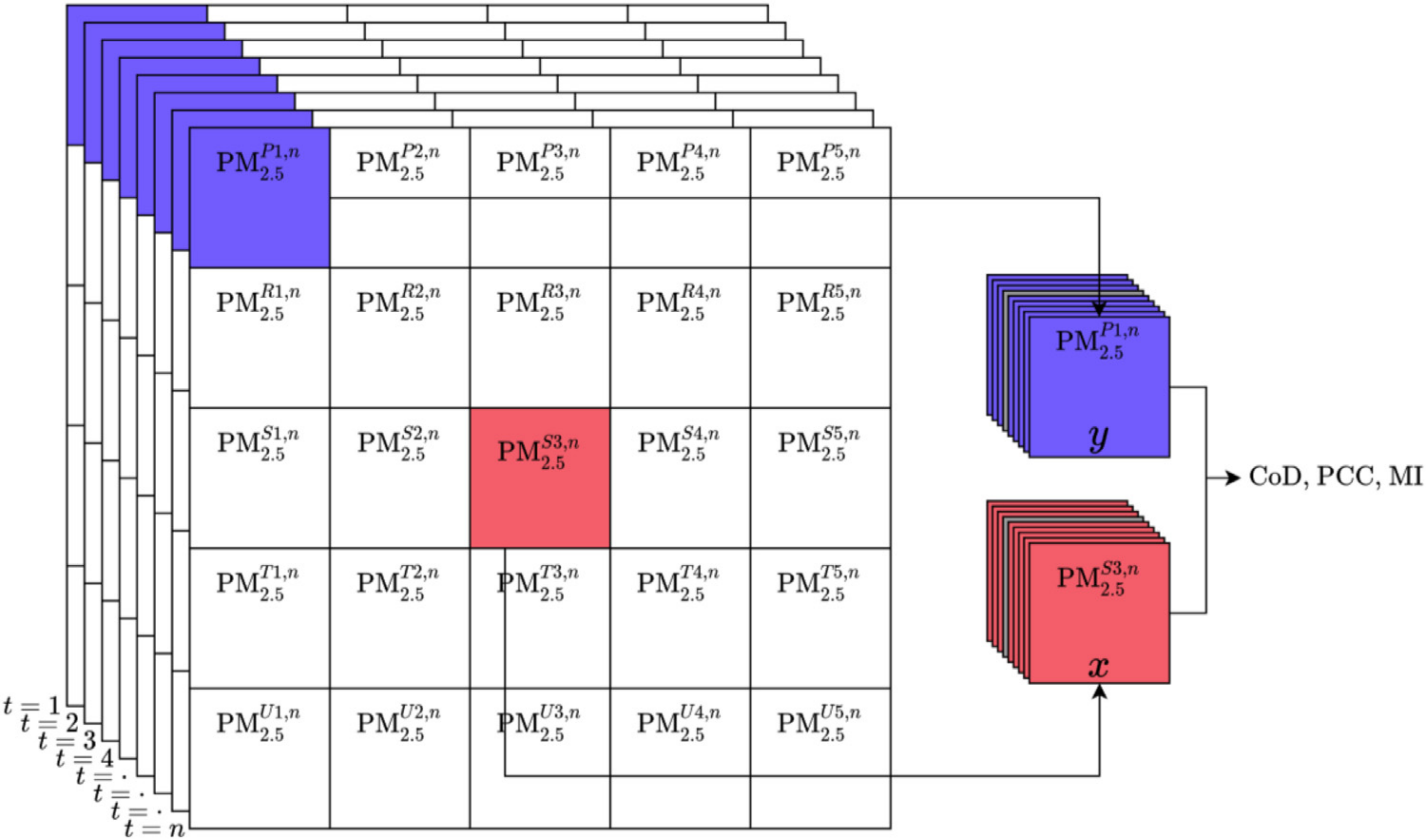
Schematic of arrangement of data and computation of the different metrics

The satellite-derived PM_2.5_ values obtained in the data pre-processing step is used for further analysis. A schematic of the PM_2.5_ data obtained is shown in Fig. 4.

Following are the important points to note in data analysis,

1. A python code namely computation.py was developed to compute the different metrics like coefficient of divergence (CoD), Pearson correlation coefficient (PCC) and mutual information (MI).
2. The reference cell (location where the sampler is planned to be placed) is identified in the data using site's latitude and longitude. For all the cells in a region of about 300 km around the site, values of the metrics are calculated sequentially when the values are available at same time instants.
3. Coefficient of Divergence (CoD) is one of the statistical techniques used to compare two values, if the values were close, the metric CoD will have value close to zero, while for completely different values, the CoD will be close to unity. The CoD for two columns of values can be calculated as,
  $$\begin{array}{c} CoD=\sqrt{\frac{1}{n}\sum_{t=1}^{n}{\left(\frac{x_{t}-y_{t}}{x_{t}+y_{t}}\right)}^{2}}\\ \left(1\right) \end{array}$$
  where, *x_t_*and *y_t_* value of PM_2.5_ at the reference cell and a neighbouring cell at *t*^th^ time instant respectively, *n* are the total number of time instants
  A function for CoD was created for all the calculations and is defined as cod_calculations in the file computation.py
4. Pearson correlation coefficient (PCC) is an addition statistical metric which is used. It can easily be accessed from Python NumPy library's corrcoef function and is used as follows,
5. While CoD compares the values, and PCC quantifies the linear relationship in the data, mutual information (MI) is used to additionally capture the non-linear relationship in the data.
6. Lekinwala et al. [5] discuss the algorithm used to calculate mutual information in detail. Mutual information function mutual_information function is part of the computation.py code.
  a. The mutual information can be computed as follows,
    $$I\left(\tilde{X},\tilde{Y}\right)=\sum_{j=1}^{N}\sum_{i=1}^{N}P\left({\tilde{x}}_{i},{\tilde{y}}_{j}\right)log\left[\frac{P\left({\tilde{x}}_{i},{\tilde{y}}_{j}\right)}{P\left({\tilde{x}}_{i}\right)P\left({\tilde{y}}_{j}\right)}\right]\Delta x\Delta y$$
  b. It requires the joint density ($P(\tilde{x},\tilde{y})$) and marginal density ($P(\tilde{x})$ and $P(\tilde{y})$) of the data under consideration to compute MI value.
  c. A Gaussian kernel is fit through the *x, y* data using gaussian_kde function in SciPy library's stats class. It can be called using stats.gaussian_kde(x, y).
  d. The fitted joint density function obtained is evaluated at 100 equispaced values in range $\tilde{x}\to \left(\mu_{\tilde{x}}-6\sigma_{\tilde{x}}\right):\Delta x:\left(\mu_{\tilde{x}}+6\sigma_{\tilde{x}}\right)$ and $\tilde{y}\to \left(\mu_{\tilde{y}}-6\sigma_{\tilde{y}}\right):\Delta y:\left(\mu_{\tilde{y}}+6\sigma_{\tilde{y}}\right)$ which is then summed across all the values of $\tilde{x}$ to obtain $P(\tilde{y})$ and vice-versa.
  e. The complete mutual_information function with appropriate comments about the lines of codes is given below,
7. The calculated values are saved as *.csv files and can be plotted to obtain spatial plots using Python code which are part of the attached computation.py file

## Data Visualisation

**Fig. 5:**
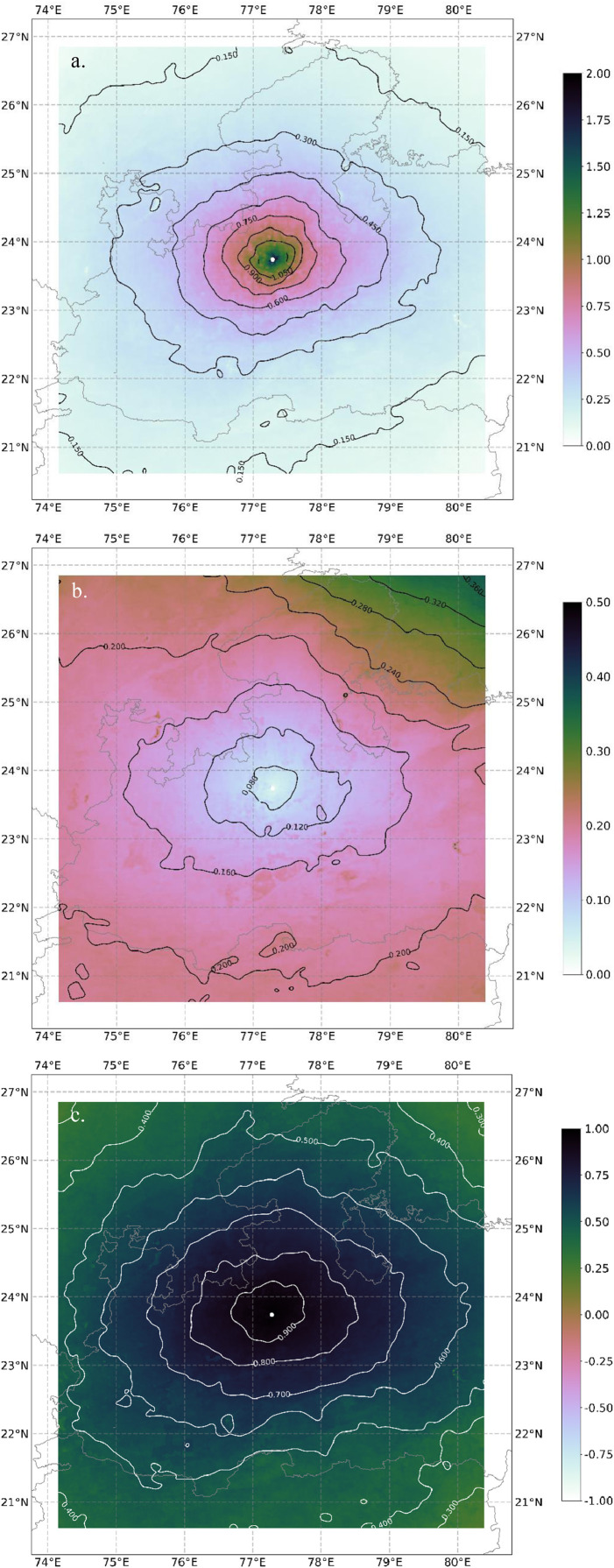
Figures generated using the aforementioned code for (a) Mutual Information, (b) Coefficient of Divergence and (c) Pearson correlation coefficient

The values of CoD, PCC and MI calculated for different sites need to be visualised to interpret their spatial characteristics. Matplotlib is a widely used plotting library in Python. Following are some important steps in plotting,

1. The saved csv file is read using Python's Pandas library using read_csv function.
2. Matplotlib's pcolormesh function is used to create a colormesh plot based on the values of metric. A cubehelix colormap is used for all the plots.
3. Additionally, contours are added to the plot to create a clear demarcation of different values. Matplotlib's contour function is used to create it.
4. Several fonts related and contour plot related options are used to make the plot visually better. Other functions and options used in the code are shown below.
5. The plots for MI (a) CoD (b) and PCC (c) created using the code in the computation.py file are presented in Fig. 5. Results and interpretation of Fig. 5 for Bhopal site and other sites are discussed in [5].

## Declaration of Competing Interest

The authors declare that they have no known competing financial interests or personal relationships that could have appeared to influence the work reported in this paper.

## Appendix Supplementary materials

Image, application 1
