# Supplementary material for: Weight-of-evidence approach to identify regionally representative sites for air-quality monitoring network: Satellite data-based analysis
Source: MethodsX. 2020 Jun 4;7:100949. doi: 10.1016/j.mex.2020.100949 (PMC7317679; doi:10.1016/j.mex.2020.100949)
Supplement: Supplementary file 1 [file mmc1.docx]

**Supplementary material:** The article is accompanied by all the codes that were developed for the work, following is a short description for each file.

1. processing_01.ncl: The file contains NCL code for pre-processing of hdf files and convert it to netCDF (nc) file.
2. rectilinear.ncl: The file contains NCL code for creating blank rectilinear grid under consideration.
3. regrid.txt: The details about the regrid/remapping of the data from 1 $\times$ 1 km to 1.5 $\times$ 1.5 km.
4. computation.py: The file contains the Python code for data analysis and data visualisations.
